# Examining the social mechanism linking excessive video gaming and depressive symptoms among adolescents: interplay of friend support and parenting quality

**DOI:** 10.1192/bjo.2025.10904

**Published:** 2025-11-17

**Authors:** Rosa S. Wong, Keith T. S. Tung, Patrick Ip

**Affiliations:** Department of Special Education and Counselling, The Education University of Hong Kong, Hong Kong SAR, China; Department of Paediatrics and Adolescent Medicine, The University of Hong Kong, Hong Kong SAR, China; Department of Paediatrics and Adolescent Medicine, Hong Kong Children’s Hospital, Hospital Authority, Hong Kong SAR, China

**Keywords:** Video gaming, depressive symptoms, peer support, parenting, adolescence

## Abstract

**Background:**

Some adolescents can achieve academic success and maintain well-being despite their engagement in video gaming. Social factors may play a role in their vulnerability to mental health problems.

**Aims:**

This study examined the role of perceived peer support and childhood experiences of optimal parenting in the association between video-gaming duration and depressive symptoms in adolescents.

**Method:**

A sample of 1071 adolescents (mean age 13.62 years, s.d. = 0.95) completed a questionnaire on video-game usage. Their perceptions of parental care and support since childhood were assessed using the Parental Bonding Instrument, whereas their perceived peer friend support was assessed using the friend support subscale of the Multidimensional Scale of Perceived Social Support. Their depressive symptoms were measured using the depression subscale of the Depression Anxiety Stress Scales. Moderated mediation analysis was conducted to examine the associations of these variables. Family socioeconomic status and symptoms of attention-deficit hyperactivity disorder were included as covariates.

**Results:**

Longer durations of video gaming were associated with higher levels of depressive symptoms. The role of perceived peer support in this association was moderated by childhood experiences of optimal parenting. Specifically, the mediating role of perceived friend support was significant only for adolescents who lacked optimal parenting.

**Conclusions:**

The relationship between frequent video gaming and depressive symptoms in adolescents is complex and may depend on the levels of peer and parental support. Lacking support from both parents and peers can increase adolescents’ risk of depression associated with frequent video gaming.

Game usage refers to the amount of time spent playing video games, including both clinical and commercial types. Video-game elements promote intrinsic motivation and enjoyment, which can sometimes contribute to excessive gaming behaviours.^1^ However, the mental health consequences of video-game engagement are complex and depend on a range of factors such as the duration and content of the games.^2^ While playing video games can provide short-term pleasure and psychological benefits, excessive gaming is partially linked to deficits in executive functions, which can impair an individual’s ability to regulate participation in reward-seeking behaviours such as gaming.^3^ This reduction in cognitive-control abilities may also contribute to the development of mental health problems, such as depression.^4^

Apart from shared cognitive risk factors, the connection between excessive video gaming and depression may operate through social deficits. The social displacement theory suggests that excessive engagement with video games may diminish opportunities for physical activity and real-life social interactions. Over time, this can lead to a social deficit pathway, whereby depressive symptoms are exacerbated through a reduction in both the quantity and quality of offline social connections.^5^ As proposed by the social support and buffering theory,^6^ there are two models – the buffering model and the main-effect model. The buffering model suggests that social relationships can buffer the negative effects of stressful events by providing affected individuals with support and care. On the other hand, the main-effect model proposes that having a large social network, such as a group of friends, benefits well-being by providing individuals with stable and socially rewarding roles within their social community. Different studies have shown the benefits of social support across different support sources in the prevention and treatment of depression.^7^

Social support refers to actions and behaviours that convey to an individual, either directly or indirectly, that they are appreciated and looked after by others. A classification system for social support outlines five key categories of support: sharing information, emotional support, feeling respected, having a social network and practical help.^8^ Of special note, online interactions are not as effective as offline interactions in providing practical help and showing respect.^9^ Furthermore, authentic support from real-life friends is essential for receiving meaningful emotional assistance.^10^ Individuals who are dissatisfied with the support they receive from offline contacts or lack satisfactory support from offline social contacts may become involved in online social interactions to seek alternative sources of support.^11^ However, a meta-analysis of studies on the relationship between online social support and adolescent mental health found that the support received from internet acquaintances may not yield the same positive impact as the support received from real-life acquaintances.^12^ When the need for relatedness is not met, individuals may experience social isolation, rejection and feelings of loneliness. It is noteworthy that real-life relationships include not just friends but also parents, who are important sources of social support. Research shows that optimal parenting involves guiding children’s screen use with reasoning and by considering their perspective.^13^ This approach can help children develop social skills, make friends and balance gaming with other activities.^14,15^

Given the potential effect of social factors, this study aimed to examine three research questions on the link between video-gaming duration and depressive symptoms in Chinese junior high school students: (a) whether frequent video gaming is significantly associated with adolescent depressive symptoms; (b) whether this association can be mediated by perceived support from friends and (c) whether this association can be moderated by childhood experiences of optimal parenting. The hypothesised model was shown in Fig. 1. Based on the theories and empirical evidence reviewed above, we expected that longer durations of video gaming were associated with higher levels of depressive symptoms (Hypothesis 1). Additionally, longer durations of video gaming were associated with lower levels of perceived support from friends, which in turn were linked to higher levels of depressive symptoms (Hypothesis 2). However, the role of friend support was moderated by childhood experiences of optimal parenting, with more pronounced peer influences observed in adolescents who lacked optimal parenting (Hypothesis 3).

**Fig. 1:**
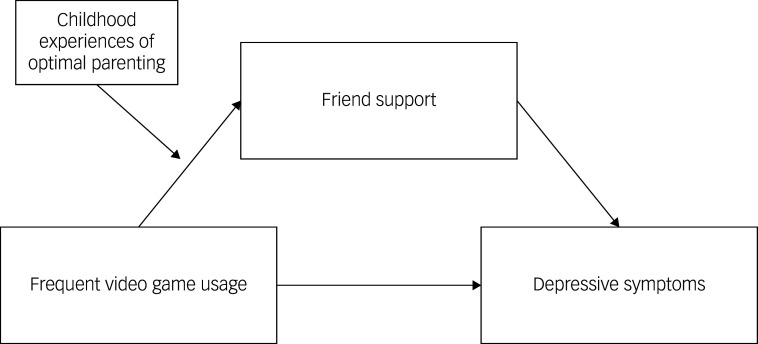
Hypothesised model.

## Method

### Participants

We recruited eligible adolescents aged 11–14 who can read Chinese or English and had no physical disabilities through local high schools, social media and adolescent community centres in Hong Kong and invited them to complete our survey either in paper format or electronically. We chose this age group because early adolescence represents a transition period marked by a gradual shift from complete reliance on parents to greater independence and peer influences. Studying this population can capture the influences of both parental support and peer support. By utilising this multi-recruitment sites approach, we were able to gather valid data from 1071 Chinese adolescents.

### Procedure

This study was approved by the Institutional Review Board of The University of Hong Kong and the Hospital Authority Hong Kong West Cluster (UW 19-722). It involved two sets of questionnaires, one for adolescents to self-report and another for parents to report on their family demographics. The online survey platform provided instructions for completing each survey and ensured anonymity. All participants provided their consent and completed the survey voluntarily, with the option to withdraw at any time. On average, the set of questionnaires took approximately 30 min to complete.

### Measures

#### Video-game usage

Participants completed a questionnaire about the amount of time they spent playing video games on handheld and/or other types of consoles on both weekdays and weekends over the past two months. The questionnaire is a reliable tool for assessing screen usage and has been used in previous studies involving children and adolescents in Hong Kong.^16–18^ Total gaming time was calculated using a weighted average formula ((2 × weekend + 5 × weekday) ÷ 7) and reported in hours.

#### Support from friends

The friend support subscale of the Multidimensional Scale of Perceived Social Support (MSPSS)^19^ was used to assess participants’ perceptions of support received from friends in the past month. The MSPSS has been widely used in studies involving Chinese adolescents and has demonstrated good reliability and validity.^20,21^ The friend support subscale comprised four items (e.g. ‘My friends really try to help me.’) rated on a 7-point Likert scale, ranging from 1 (strongly disagree) to 7 (strongly agree). Higher scores indicated higher levels of support from friends. In our study, Cronbach’s *α* for this scale was 0.93.

#### Childhood experiences of optimal parenting

The Parental Bonding Instrument (PBI)^22^ was used to assess participants’ perceptions of parental care and support during their childhood. The Chinese version of the PBI has been validated in Hong Kong and shown to be valid and reliable.^23,24^ The PBI consists of two subscales: one evaluates care (12 items) and the other evaluates overprotection (13 items). Participants responded to these items separately for their mothers and fathers using a 4-point Likert scale, ranging from 0 (very unlike) to 3 (very like). Higher scores indicated a greater inclination towards the measured parenting practice. Furthermore, we categorised their mothers and fathers into ‘high’ and ‘low’ groups based on the specific subscale cut-off scores (care score of 27 for mothers and 24 for fathers, and overprotection score of 13.5 for mothers and 12.5 for fathers).^22^ Optimal parenting was defined as having high care and low overprotection. Participants whose mothers and fathers did not meet the criteria were considered to have suboptimal parenting in subsequent analyses. In our study, Cronbach’s *α* for the care scale was 0.85 for the mother and 0.88 for the father. Additionally, Cronbach’s *α* for the overprotection scale was 0.78 for the mother and 0.80 for the father.

### Depressive symptoms

The depression subscale of the Depression Anxiety Stress Scale (DASS-21)^25^ was used to measure levels of depressive symptoms in participants at the time of completing the scale. DASS-21 is a suitable assessment tool for Chinese adolescents as it has been proven to be both reliable and valid.^26,27^ The depression subscale included 7 items (e.g. ‘I found it difficult to work up the initiative to do things’) rated on a 4-point Likert scale, ranging from 0 (strongly disagree) to 3 (strongly agree). The total score was generated by adding the item scores and then multiplying the sum by 2. Higher scores indicated more depressive symptoms. In our study, Cronbach’s *α* for this scale was 0.81.

### Covariates

Since previous studies have demonstrated significant associations of attention-deficit hyperactivity disorder (ADHD) with screen time and depression,^28–30^ adjusting for ADHD symptoms can minimise the likelihood of a false association between video-gaming duration and depressive symptoms in participants. Specifically, we asked their primary caregiver to complete the Chinese version of the Strengths and Weaknesses of ADHD Symptoms and Normal Behaviour Scale (SWAN), which has been validated in Hong Kong.^31^ This scale consists of 18 items rated on a 7-point Likert scale, with values ranging from −3 (far above normal) to +3 (far below normal). The total score, obtained by summing individual item scores, indicates the severity of ADHD symptoms in participants. Additionally, the primary caregiver provided their educational and employment details, as well as their spouse’s, along with their monthly household income. These demographic details were combined to generate a socioeconomic status (SES) index. This index was calculated using principal component analysis with varimax rotation, with greater values indicating higher social classes.^32^ Participants’ age and gender were collected through self-report.

### Data analysis

Data analysis was performed using the software package SPSS 22.0. To summarise participants’ sociodemographic characteristics, we computed descriptive statistics, including the mean and s.d. for continuous variables, and frequencies and percentages for categorical variables. Pearson’s correlation analyses were conducted to examine the relationships between the variables of interest. Additionally, we employed model 4 of the PROCESS macro to examine the mediating role of perceived friend support between video-gaming duration and depressive symptoms. To explore the role of childhood experiences of optimal parenting in these associations, we utilised model 7 of the PROCESS macro, which is commonly used for testing moderated mediation models. To assess the significance of conditional direct and indirect effects, we estimated bias-corrected bootstrap confidence intervals using 5000 bootstrap resamples. A significant effect was indicated by a confidence interval that did not include zero.

## Results

### Descriptive statistics and correlations among the variables

Table 1 provides descriptive statistics. The mean age of the 1071 participants (471 females and 600 males) was 13.62 years old, with an s.d. of 0.95. Around 30% of their fathers and mothers had completed tertiary education. Additionally, a majority of fathers (87.7%) were engaged in full-time employment, while a substantial proportion of mothers (40.7%) were housewives. Moreover, 559 (52.2%) families had a monthly household income that fell below the median income in Hong Kong (HKD 30 000). Correlation analyses revealed that depressive symptoms were associated negatively with support from friends (*r* = −0.26, *p* < 0.001) and positively with ADHD symptoms (*r* = 0.19, *p* < 0.001), whereas support from friends was negatively associated with ADHD symptoms (*r* = −0.14, *p* < 0.001).

**Table 1:** Participant characteristics

Characteristics	Overall (*n* = 1071)
Age, mean (s.d.)	13.62 (0.95)
Gender, *n* (%)	
Female	471 (44.0)
Male	600 (56.0)
Monthly household income^a^, *n* (%)	
<HKD 30 000	559 (52.2)
≥HKD 30 000	509 (47.5)
Mother	
Education level^a^, *n* (%)	
Primary education	49 (4.6)
Secondary education	690 (64.6)
Tertiary education	329 (30.8)
Job status^a^, *n* (%)	
Full-time	476 (44.9)
Part-time	138 (13.0)
Unemployed	15 (1.4)
Homemaker	432 (40.7)
Father	
Education level^a^, *n* (%)	
Primary education	50 (4.8)
Secondary education	648 (62.7)
Tertiary education	335 (32.4)
Job status^a^, *n* (%)	
Full-time	889 (87.7)
Part-time	48 (4.7)
Unemployed	46 (4.5)
Homemaker	31 (3.1)
Optimal parenting, *n* (%)	
None	621 (58.0)
Have at least one optimal parent	450 (42.0)
Depressive symptoms, mean (s.d.)	7.92 (7.74)
Support from friends, mean (s.d.)	20.91 (9.58)
ADHD symptoms, mean (s.d.)	−0.39 (0.99)

ADHD, attention-deficit hyperactivity disorder.

a. Missing data not shown.

Based on the recommended cut-off score of 21 on the DASS depression scale,^26,27^ 79 participants (7.4%) were identified as having significant depressive symptoms. Compared to their counterparts, these participants reported significantly lower levels of friend support (16.86 *v*. 21.24, *p* < 0.001), longer durations of video gaming (4.71 *v*. 3.97, *p* = 0.016), reduced perceived maternal care (19.89 *v*. 25.73, *p* < 0.001) and lower paternal care (17.91 *v*. 23.95, <0.001). Additionally, they perceived higher maternal overprotection (16.39 *v*. 13.34, *p* < 0.001) and paternal overprotection (14.32 *v*. 11.34, *p* < 0.001).

### Testing for mediation

As shown in Table 2, after adjusting for age, gender, family SES and ADHD symptoms, video-game usage was positively associated with depressive symptoms (*β* = 1.31, *p* = 0.006) and depressive symptoms were negatively associated with support from friends (*β* = −0.34, *p* < 0.001). The association of video-game usage with depressive symptoms remained significant after further adjusting for support from friends (*β* = 1.14, *p* = 0.014). However, video-game usage was not associated with support from friends (*β* = −0.53, *p* = 0.127). Therefore, support from friends did not mediate the relationship between video-game usage and depressive symptoms before taking parenting influences into account.

**Table 2:** Testing the effect of video-game usage on depressive symptoms via support from friends

Outcome	Predictors	*R*^2^	*F*	*β*	Low limit CI	Upper limit CI	*t* value
Dep	Age	0.06	12.84	0.18	−0.3	0.66	0.75
	Gender			1.99	1.05	2.92	4.15***
	FSES			0.04	−0.23	0.3	0.26
	ADHD symptoms			1.59	1.12	2.06	6.67***
	VGU			1.31	0.39	2.24	2.78**
Support from friends	Age	0.03	7.71	−0.09	−0.44	0.26	−0.51
	Gender			1.1	0.42	1.78	3.16**
	FSES			0.1	−0.09	0.29	1.01
	ADHD symptoms			−0.71	−1.05	−0.37	−4.08***
	VGU			−0.53	−1.2	0.15	−1.53
Dep	Age	0.11	22.77	0.15	−0.31	0.62	0.64
	Gender			2.36	1.44	3.27	5.06***
	FSES			0.07	−0.19	0.32	0.52
	ADHD symptoms			1.35	0.89	1.81	5.79***
	VGU			1.14	0.24	2.04	2.47*
	Support from friends			−0.34	−0.42	−0.26	−8.27***

*N* = 1071, Dep, depressive symptoms; FSES, family socioeconomic status; ADHD, attention-deficit hyperactivity disorder; VGU, video-game usage. Bootstrap sample size = 5000. The beta coefficient was unstandardised. *p < 0.05, **p < 0.01, ***p < 0.001.

### Testing for moderated mediation

Hypothesis 3 predicted that perceived suboptimal care and overprotection from parents during childhood would strengthen the association between frequent video gaming and depressive symptoms via reduced support from friends. As shown in Table 3, after adjusting for age, gender, family SES and ADHD symptoms, a significant interaction effect of video-game usage and childhood experiences of optimal parenting on support from friends was observed (*β* = −1.66, *p* = 0.014). Simple slope tests showed that the association between video-game usage and support from friends was significant only in adolescents who experienced suboptimal parenting during childhood (*β* = −1.12, *p* = 0.011), but not in those who perceived optimal care and support from at least one parent (*β* = 0.55, *p* = 0.297). That is, the association between frequent video gaming and depressive symptoms through reduced support from friends was significant only when adolescents lacked optimal care and support from both parents during childhood (indirect effect 0.38, s.e. = 0.16, 95% CI = 0.08–0.72). Therefore, Hypothesis 3 was supported.

**Table 3:** Testing the moderated mediation effect of video-game usage on depressive symptoms

Outcome	Predictors	*R* ^2^	*F*	*β*	Low limit 95% CI	Upper limit 95% CI	*t* value
Support from friends	Age	0.07	11.3	−0.12	−0.46	0.23	−0.67
	Gender			1.25	0.58	1.92	3.63***
	FSES			0.09	−0.1	0.27	0.9
	ADHD symptoms			−0.57	−0.91	−0.22	−3.26**
	VGU			2.21	0.003	4.41	1.96*
	LOP			0.53	−1.56	2.61	0.49
	VGU x LOP			−1.66	−2.99	−0.34	−2.47*
Dep	Age	0.11	22.77	0.15	−0.31	0.62	0.64
	Gender			2.36	1.44	3.27	5.06***
	FSES			0.07	−0.19	0.32	0.52
	ADHD symptoms			1.35	0.89	1.81	5.79***
	VGU			1.14	0.24	2.04	2.47*
	Support from friends			−0.34	−0.42	−0.26	−8.27***
Conditional effect of VGU on support from friends at the values of the moderator:	*β*	Boot s.e.	Boot low limit 95% CI	Boot upper limit 95% CI			
Have at least one optimal parent			0.55	0.52	−0.48	1.57	
Lack of optimal parenting			−1.12	0.44	−1.98	−0.26	
Conditional indirect-effect analysis at the values of the moderator:	*β*	Boot s.e.	Boot low level 95% CI	Boot upper limit 95% CI			
Have at least one optimal parent			−0.18	0.18	−0.55	0.17	
Lack of optimal parenting			0.38	0.16	0.08	0.72	

*N* = 1071, Dep, depressive symptoms; FSES, family socioeconomic status; ADHD, attention-deficit hyperactivity disorder; VGU, video game usage; LOP, lack of optimal parenting. Bootstrap sample size = 5000. The beta coefficient was unstandardised. *p < 0.05, **p < 0.01, ***p < 0.001.

## Discussion

The findings of this study indicate the amount of time spent playing video games as a significant lifestyle factor associated with depressive symptoms in adolescents. We found that adolescents who spent more time playing video games were more likely to experience depressive symptoms. Additionally, perceived support from friends served as a conditional mediator between frequent video gaming and depressive symptoms. The significance of peer support was moderated by childhood experiences of optimal parenting, with more pronounced peer influences observed in adolescents who lacked optimal care and support from both parents during childhood. Nonetheless, perceived support from friends, when not stratified by childhood experiences of optimal parenting, did not mediate the association between frequent video gaming and adolescent depressive symptoms. These findings highlight the significance of parenting influences, which can counteract the negative effect of reduced peer support on adolescent mental health. However, the development of depression is complex, involving various biological, cognitive, emotional and interpersonal risk factors.^33^ Future research should consider exploring other pathways through which video-game usage could potentially influence lifestyle habits and how these lifestyle changes relate to depressive symptoms. For example, the social displacement theory^5^ suggests that increased gaming time may encroach on opportunities for healthy activities, such as physical exercise. Furthermore, excessive gaming can lead to cognitive overstimulation, which may impair sleep quality and potentially exacerbate depression.^34,35^

We found evidence for the association between frequent video gaming and depressive symptoms through reduced peer support but only for adolescents who lacked optimal care and support from both parents during childhood. In other words, adolescents who grow up in a neglectful or overprotective family environment may be more susceptible to depression when they concurrently experience insufficient peer support due to excessive video gaming. Our results align with the principles of basic psychological need theory,^36^ which emphasise the importance of relatedness for maintaining health and well-being. Previous research has shown that real-life social support has a greater positive impact on mental health than support received through social media.^10^ Excessive gaming may reduce opportunities to foster feelings of relatedness and belonging in real-world relationships. Our findings also demonstrate that increased time spent playing video games may adversely influence adolescents’ perceptions of peer support, particularly when parental support is lacking, potentially undermining their mental health.

According to Bronfenbrenner’s bioecological theory,^37^ an individual’s growth and development occur in distinct patterns that are shaped by the interplay of protective and risk exposures within and across different ecological levels. These exposures, either alone or in combination, can have an impact on health outcomes and developmental trajectories. Consistent with this perspective, our findings show that suboptimal interpersonal factors, such as a lack of support from both parents and friends, combined with suboptimal lifestyle choices such as frequent video gaming, can increase negative emotions. Previous research has identified certain individual factors, such as neuroticism, that may elevate the risk of developing depressive symptoms.^38^ It is plausible that positive environmental factors, such as supportive surroundings, can mitigate the risks associated with suboptimal behavioural choices or innate characteristics to adolescent mental health. For example, optimal parenting was found to be a protective factor in this study, which aligns with previous research showing that caring and understanding parenting approaches can promote children’s social skills, such as children’s ability to accurately interpret emotions and navigate social interactions in a positive direction.^39^ There is also evidence suggesting that adolescents with higher levels of parental support are less likely to experience emotional problems due to a lack of support from another source.^40^

It is worth noting that the mediating role of peer support was significant only for participants who experienced suboptimal parenting during childhood. Our study did not examine whether and how frequent video gaming may influence adolescent mental health in a supportive family context. Notably, some studies showed that individuals who heavily engage in video games may struggle with self-control issues, which can be partly attributed to changes in brain structure associated with depression.^4^ These cognitive problems can be inborn or caused by factors outside of the family context and warrant further investigation. The results of this study have several practical implications. First, interventions should prioritise reducing problematic video gaming. Due to the widespread availability of video gaming, it can be challenging to completely abstain from it. Therefore, interventions can focus on moderating gaming use and increasing participation in non-technology or offline activities. Second, given that perceived peer support is a conditional mediator linking video gaming to depressive symptoms, we should promote offline social activities for high game players to enhance their sense of peer support. For instance, mentorship programmes can be established where experienced high game players can offer guidance and support to newer or struggling players. This can help to improve relationships between players and provide opportunities for peer support that extend beyond the virtual gaming world. Third, the moderating effect of parenting quality indicates that proper parenting strategies, such as showing higher levels of care and avoiding excessive overprotection, provide crucial social resources to mitigate the social deficit pathway that connects video gaming to depressive symptoms.

Several limitations should be considered. First, this study relied on a cross-sectional survey design, making it impossible to establish causal associations. Future research could benefit from longitudinal data or experimental studies to better ascertain the findings. It is also important to investigate whether the association between gaming and depression is bidirectional and driven by the same underlying mechanisms. Second, while the self-report method is commonly used in literature to measure depressive symptoms, future studies should consider employing multiple methods to gather data from various sources including parents and peers. Third, caution should be exercised when generalising our findings from early adolescents to other age groups. Furthermore, while our results were adjusted for ADHD symptoms, other unmeasured neurodevelopmental problems, such as autism, may also have an impact on the findings. Last, this study examined perceived support from friends and parents among participants who are predominantly below the clinical depression threshold. Other factors such as school connectedness and loneliness may also influence the association. Future research should investigate how the severity of gaming behaviour, such as addiction, is linked to clinical depression, whether in a manner similar to or different from the findings of this study.

In conclusion, this study sheds light on a social deficit pathway through which frequent video gaming is linked to depressive symptoms in early adolescents. Support from friends and parents plays a role in the association between frequent video gaming and depressive symptoms in adolescents. Parental support can mitigate the negative effect of frequent video gaming on perceived peer support and depressive symptoms in adolescents. Promoting peer support is conducive to mitigating depressive symptoms particularly for adolescent gamers who lack optimal parenting.

## Data Availability

The data that support the findings of this study are available on request from the corresponding author.

## References

[1] Arbeau K , Thorpe C , Stinson M , Budlong B , Wolff J. The meaning of the experience of being an online video game player. Comput Human Behav Rep 2020; 2: 100013.

[2] Hartanto A , Lua VYQ , Quek FYX , Yong JC , Ng MHS. A critical review on the moderating role of contextual factors in the associations between video gaming and well-being. Comput Human Behav Rep 2021; 4: 100135.

[3] Dong G , Potenza MN. A cognitive-behavioral model of Internet gaming disorder: theoretical underpinnings and clinical implications. J Psychiatr Res 2014; 58: 7–11. 25062755 10.1016/j.jpsychires.2014.07.005 PMC4448942

[4] Dotson VM , McClintock SM , Verhaeghen P , Kim JU , Draheim AA , Syzmkowicz SM , et al. Depression and cognitive control across the lifespan: a systematic review and meta-analysis. Neuropsychol Rev 2020; 30: 461–76. 32385756 10.1007/s11065-020-09436-6 PMC9637269

[5] Kowert R , Domahidi E , Festl R , Quandt T. Social gaming, lonely life? The impact of digital game play on adolescents’ social circles. Comput Human Behav 2014; 36: 385–90.

[6] Cohen S , Wills TA. Stress, social support, and the buffering hypothesis. Psychol Bull 1985; 98: 310–57. 3901065

[7] Rueger SY , Malecki CK , Pyun Y , Aycock C , Coyle S. A meta-analytic review of the association between perceived social support and depression in childhood and adolescence. Psychol Bull 2016; 142: 1017. 27504934 10.1037/bul0000058

[8] Ko HC , Wang LL , Xu YT. Understanding the different types of social support offered by audience to A-list diary-like and informative bloggers. Cyberpsychol Behav Soc Network 2013; 16: 194–9. 10.1089/cyber.2012.0297 PMC3603495 23363225

[9] Liu D , Wright KB , Hu B. A meta-analysis of social network site use and social support. Comput Educ 2018; 127: 201–13.

[10] Meshi D , Ellithorpe ME. Problematic social media use and social support received in real-life versus on social media: associations with depression, anxiety and social isolation. Addict Behav 2021; 119: 106949. 33934007 10.1016/j.addbeh.2021.106949

[11] Chung JE. Social interaction in online support groups: preference for online social interaction over offline social interaction. Comput Human Behav 2013; 29: 1408–14.

[12] Zhou Z , Cheng Q. Relationship between online social support and adolescents’ mental health: a systematic review and meta-analysis. J Adolesc 2022; 94: 281–92. 35390193 10.1002/jad.12031

[13] Detnakarintra K , Trairatvorakul P , Pruksananonda C , Chonchaiya W. Positive mother-child interactions and parenting styles were associated with lower screen time in early childhood. Acta Paediatr 2020; 109: 817–26. 31509278 10.1111/apa.15007

[14] Goering M , Mrug S. Empathy as a mediator of the relationship between authoritative parenting and delinquent behavior in adolescence. J Youth Adolesc 2021; 50: 1308–18. 33991274 10.1007/s10964-021-01445-9 PMC8343943

[15] Goagoses N , Bolz T , Eilts J , Schipper N , Schütz J , Rademacher A , et al. Parenting dimensions/styles and emotion dysregulation in childhood and adolescence: a systematic review and meta-analysis. Curr Psychol 2023; 42: 18798–822.

[16] Wong RS , Tung KTS , Rao N , Leung C , Hui ANN , Tso WWY , et al. Parent technology use, parent–child interaction, child screen time, and child psychosocial problems among disadvantaged families. J Pediatr 2020; 226: 258–65. 32629010 10.1016/j.jpeds.2020.07.006

[17] Wong CK , Wong RS , Cheung JP , Tung KT , Yam JC , Rich M , et al. Impact of sleep duration, physical activity, and screen time on health-related quality of life in children and adolescents. Health Qual Life Outcomes 2021; 19: 145. 33980245 10.1186/s12955-021-01776-y PMC8117552

[18] Wong RS , Tung KTS , Rao N , Ho FKW , Chan KL , Fu K-W , et al. A longitudinal study of the relation between childhood activities and psychosocial adjustment in early adolescence. Int J Environ Res Public Health 2021; 18: 5299. 34065751 10.3390/ijerph18105299 PMC8157182

[19] Zimet GD , Dahlem NW , Zimet SG , Farley GK. The multidimensional scale of perceived social support. J Personal Assess 1988; 52: 30–41.

[20] Wong RS , Tung KTS , Fu K-W , Bacon-Shone J , Molasiotis A , Li WO , et al. Examining social context and the pathways to mental wellness in young adults during social movement: a parallel mediation analysis. J Affect Disord 2021; 294: 876–82. 34375215 10.1016/j.jad.2021.07.100

[21] Wan L-P , Yang X-F , Liu B-P , Zhang Y-Y , Liu X-C , Jia C-X , et al. Depressive symptoms as a mediator between perceived social support and suicidal ideation among Chinese adolescents. J Affect Disord 2022; 302: 234–40. 35090945 10.1016/j.jad.2022.01.061

[22] Parker G , Tupling H , Brown LB. A parental bonding instrument. Br J Med Psychol 1979; 52: 1–10.

[23] Ngai SS-y , Cheung C-k , Xie L , Ng Y-h , Ngai H-l , Liu Y , et al. Psychometric properties of the parental bonding instrument: data from a Chinese adolescent sample in Hong Kong. J Child Family Stud 2018; 27: 2112–24.

[24] Wong RS , Tung KTS , Chan KL , Wong WHS , Tsang HW , Chow CHY , et al. Evidence of individual differences in the long-term social, psychological, and cognitive consequences of child maltreatment. Child Adolesc Psychiatry Ment Health 2022; 16: 88. 36424655 10.1186/s13034-022-00524-4 PMC9686092

[25] Lovibond SH , Lovibond PF. Manual for the Depression Anxiety Stress Scales 2nd ed. Psychology Foundation, 1995.

[26] Mellor D , Vinet EV , Xu X , Mamat NHB , Richardson B , Román F. Factorial invariance of the DASS-21 among adolescents in four countries. Eur J Psychol Assess 2015; 31: 138–42.

[27] Chan SKW , Chan KT , Chen EYH , Hui CLM , Lee EHM , Suen YN , et al. Excessive fear of clusters of holes, its interaction with stressful life events and the association with anxiety and depressive symptoms: large epidemiological study of young people in Hong Kong. BJPsych Open 2023; 9: e151. 37577835 10.1192/bjo.2023.540 PMC10594086

[28] Eirich R , McArthur BA , Anhorn C , McGuinness C , Christakis DA , Madigan S. Association of screen time with internalizing and externalizing behavior problems in children 12 years or younger: a systematic review and meta-analysis. JAMA Psychiatry 2022; 79: 393–405. 35293954 10.1001/jamapsychiatry.2022.0155 PMC8928099

[29] Koncz P , Demetrovics Z , Takacs ZK , Griffiths MD , Nagy T , Király O. The emerging evidence on the association between symptoms of ADHD and gaming disorder: a systematic review and meta-analysis. Clin Psychol Rev 2023; 106: 102343. 37883910 10.1016/j.cpr.2023.102343

[30] Dardani C , Davey Smith G , Leppert B , O’Donovan MC , Rice F , Riglin L , et al. ADHD and depression: investigating a causal explanation. Psychol Med 2021; 51: 1890–7. 32249726 10.1017/S0033291720000665 PMC8381237

[31] Lai KYC , Leung PWL , Luk ESL , Wong ASY , Law LSC , Ho KKY. Validation of the Chinese strengths and weaknesses of ADHD-symptoms and normal-behaviors questionnaire in Hong Kong. J Attent Disord 2011; 17: 194–202. 10.1177/1087054711430711 22210800

[32] Vyas S , Kumaranayake L. Constructing socio-economic status indices: how to use principal components analysis. Health Policy Plann 2006; 21: 459–68. 10.1093/heapol/czl029 17030551

[33] Reivich K , Gillham JE , Chaplin TM , Seligman MEP. From helplessness to optimism: the role of resilience in treating and preventing depression in youth. In Handbook of Resilience in Children (eds S Goldstein , RB Brooks ): 161–74. Springer International Publishing, 2023.

[34] Jiang M , Zhao Y , Wang J , Hua L , Chen Y , Yao Y , et al. Serial multiple mediation of the correlation between internet addiction and depression by social support and sleep quality of college students during the COVID-19 epidemic. Psychiatry Investig 2022; 19: 9–15. 10.30773/pi.2021.0147 PMC8795596 34986557

[35] Swann OG , Kilpatrick M , Breslin M , Oddy WH. Dietary fiber and its associations with depression and inflammation. Nutr Rev 2019; 78: 394–411. 10.1093/nutrit/nuz072 31750916

[36] Vansteenkiste M , Ryan RM , Soenens B. Basic psychological need theory: advancements, critical themes, and future directions. Motiv Emot 2020; 44: 1–31.

[37] Bronfenbrenner U. Toward an experimental ecology of human development. Am Psychol 1977; 32: 513–31.

[38] Ono Y , Takaesu Y , Nakai Y , Ichiki M , Masuya J , Kusumi I , et al. The influence of parental care and overprotection, neuroticism and adult stressful life events on depressive symptoms in the general adult population. J Affect Disord 2017; 217: 66–72. 28391110 10.1016/j.jad.2017.03.058

[39] Zimmer-Gembeck MJ , Rudolph J , Kerin J , Bohadana-Brown G. Parent emotional regulation: a meta-analytic review of its association with parenting and child adjustment. Int J Behav Dev 2021; 46: 63–82.

[40] Lyell KM , Coyle S , Malecki CK , Santuzzi AM. Parent and peer social support compensation and internalizing problems in adolescence. J School Psychol 2020; 83: 25–49. 10.1016/j.jsp.2020.08.003 33276854

